# Quality of life, depression and dietary intake in Obstructive Sleep Apnea patients

**DOI:** 10.1186/s12955-016-0516-5

**Published:** 2016-07-27

**Authors:** Marta Stelmach-Mardas, Marcin Mardas, Khalid Iqbal, Robert J. Tower, Heiner Boeing, Tomasz Piorunek

**Affiliations:** 1 Department of Epidemiology, German Institute of Human Nutrition Potsdam-Rehbruecke, Arthurt-Scheunert Alee Str. 114-116, 14558 Nuthetal, Germany; 2 Department of Pediatric Gastroenterology and Metabolic Diseases, Poznan University of Medical Sciences, Szpitalna Str 27/33, 60-572 Poznan, Poland; 3 Department of Human Nutrition and Hygiene, Poznan University of Life Sciences, Wojska Polskiego Str. 28, 60-637 Poznań, Poland; 4 Department of Development and Regeneration, Laboratory of Skeletal Cell Biology and Physiology, Skeletal Biology and Engineering Research Center, O&N 1 Herestraat 49 box 813, KU Leuven, Leuven, Belgium; 5 Department of Pulmonology, Allergology and Respiratory Oncology, Poznan University of Medical Sciences, Szamarzewskiego Str. 84, 60-569 Poznań, Poland

**Keywords:** Sleep Apnea, Chronotype, Diet, Biomarkers

## Abstract

**Background:**

The aim of this study was to analyze the association between depression, quality of life and dietary intake in newly diagnosed Obstructive Sleep Apnea (OSA) patients.

**Methods:**

From 153 eligible patients suffering from sleep disturbances, 64 met inclusion and exclusion criteria. The polysomnography was used for OSA diagnosis. The quality of life (QOL) was assessed by WHOQOL-BREF questionnaire, self-reported chronotype by morningness-eveningness questionnaire and level of depression by Beck’s Depression Inventory. Blood pressure and parameters of glucose and lipid metabolism were assessed by routine methods. The dietary intake was evaluated by 24-hr dietary recalls.

**Results:**

Significantly negative associations were found between depression inventory and QOL. Better QOL for physical health and social relationships was observed in the “*definitely morning”* chronotype. The *“morning type”* of patients was positively related to the intake of fat, monounsaturated fatty acids and vitamin B12. Correlations between QOL and diastolic blood pressure, HDL-cholesterol, TG, fasting glucose, as well as protein and vitamin B6 intake were found.

**Conclusions:**

In conclusion, both chornotype and depression influence QOL in OSA patients where morning type is associated with better physical health and social relationships and increase in depression index deteriorate physical health, psychological and social relationship QOL domains. QOL as well as depression and chornotype are also influenced by selected cardio-metabolic factors and dietary intake.

## Background

Obstructive Sleep Apnea (OSA) is characterized by repetitive partial or complete closure of the upper airway during sleep that results in hypoxemia and hypercapnia, frequently associated with arousals [1]. OSA is potentially related to daytime sleepiness and different comorbidities e.g. hypertension, insulin-resistance and obesity [2–4]. Moreover, the diagnoses of depression is also emerging in OSA patients since the early 2000s. However, the relationship between such symptoms and objectively-rated features of OSA is still poorly understood [5, 6].

In addition, OSA negatively affects quality of life (QOL) and increases the risk of other comorbidities [7]. Since health related QOL in patients is recognized as an important part of diseases processes in terms of disease diagnosis and treatment success, an assessment of QOL in OSA patients is of considerable interest to improve treatment outcomes [8]. Breathing-related sleep disordered are usually associated with a poorer QOL - especially in social, emotional and physical domains [9]. Nevertheless, there are still some inconsistencies in regards to the relationship between sleepness, mood and QOL. It has been even reported in the cohort study that individuals with mild OSA do not have worse sleepiness, mood or quality of life in comparison to those without OSA [10]. QOL may also be affected by individuals’ chronotype, i.e. morning or evening type, and may be important to consider for management of OSA.

Furthermore, while the unique importance of lifestyle factors in OSA patients has already been emphasized [11, 12], there is still insufficient evidence regarding dietary intake and its association with QOL and depression in this group of patients, which may be important to include in successful OSA treatment regimes.

For successful treatment of OSA, it is critical to generate evidence regarding whether chronotype, quality of life, depression and nutrient intake are of importance in this population..This study was planned to address these questions with the specific aims to evaluate the association of chronotype and depression with quality of life, as well as to assess the correlation of cardio-metabolic risk factors and nutrients intake with QOL domains, depression and chronotype.

## Methods

### Study design and patient population

This was a cross-sectional study. We recruited patients which were admitted to the Department of Pulmonology, Allergology and Respiratory Oncology at the Poznan University of Medical Sciences in Poland for diagnostics of sleep disturbances (suspected OSA).

Inclusion criteria were as follows: age above 18, signs of OSA (snoring, apnea during sleep, morning tiredness, increased daytime sleepiness etc.), willingness to participate in the study, being on habitual diet during the period of examination. Exclusion criteria included: pregnancy or lactation, cancer (excluding curatively treated with no evidence of diseases for 5 years), severe liver and kidney diseases, diagnosed cardiovascular diseases (myocardial infarct, stroke, angina pectoris). An active drug or alcohol abuse, legal incompetence and limited legal competence were additional exclusion criteria.

One hundred fifty three patients were screened for OSA. Sixty four individuals met above criteria and were included in the study. Written informed consent was obtained from all the subjects who agreed to participate in the study. Medical history, comorbidities, concomitant medications were recorded in electronic database. Experimental protocol was approved by Bioethical Committee (400/15) at Poznan University of Medical Sciences.

### Anthropometry appraisals, assessment of circadian rhythms and depression inventory

The anthropometrical parameters included body weight and height (Seca digital scale 763, US). Height was measured using a stadiometer (with an accuracy of ±0.5 cm), and weight using a digital scale (with an accuracy of ±0.1 kg), while wearing light clothing and without shoes. Body Mass Index (BMI) was calculated to determine the degree of obesity in the study groups [13].

The polysomnography (PSG) was used as the standard method for OSA diagnosis (Embla 4500, Inc, by the Beth Israel Deaconess Medical). *The American Academy of Sleep Medicine (AASM)* recommendations [14] regarding filters, sample signal rates and configurations were followed. Flow tracing was provided by a nasal cannula and thermistor, thoracoabdominal motion by piezoelectric bands. Oxygen saturation was measured with a pulse oximeter. The apnea hypopnea index (AHI) was defined as a total number of apnea and hypopnea events divided by the total sleep time. Apnea was noted at the cessation of airflow for at least 10 s and whereas hypopnea was reported as ≥80 % reduction in airflow for at least 10 s (taking into account an amplitude) [15]. According to the AHI, OSA was classified into mild (5.0–14.9), moderate (15.0–29.9), and severe (≥30.0 events per hour) [16]. Oxygen desaturation Index (ODI) was defined as a number of significant episodes of desaturation per hour of sleep (∆ > 4 %) [15]. The electroencephalogram (EEG) was used as the primary variable to document wakefulness, arousals and sleep stages during the sleep study. PSG was analyzed by an experienced pulmonologist (TP).

### Morningness - eveningness questionnaire

All patients completed the morningness - eveningness questionnaire to assess morningness-eveningness in human circadian rhythms. The questionnaire consists of 19 questions, each with a number of points. Total scores can range from 16 to 86. Scores of 41 and below indicated “evening types”, scores of 59 and above - “morning types” and scores between 42 and 58 indicated “intermediate types” [17].

### The Beck’s depression inventory

To assess the self-reported level of depression, we used Beck’s depression inventory. It consisted of 21 questions, each with a number of points with a possible total score 63. Total scores of 1–10 was considered as normal, 11–16 as moderate mood disturbances, 17–20 as borderline clinical depression, 21–30 as moderate depression, and 31–40 indicate severe depression [18].

### Assessment of quality of life

WHO Quality of Life-BREF (WHOQOL-BREF) was used as a shorter version of the original instrument (WHOQOL-100) to assess the quality of life of OSA patients. It comprises 26 items which measure the following broad domains including selected facts: physical health, (activities of daily living, dependence on medicinal substances and medical aids, energy and fatigue, mobility, pain and discomfort, sleep and rest, work capacity) psychological health (body image and appearance, negative feelings, positive feelings, self-esteem spirituality/religion/personal beliefs, thinking, learning, memory and concentration), social relationships (personal relationships, social support, sexual activity), and environment (financial resources, freedom, physical safety and security, health and social care: accessibility and quality, home environment, opportunities for acquiring new information and skills, participation in and opportunities for recreation/leisure activities, physical environment, transport). There were also two items that were examined separately: question 1 asked about patient’s overall perception of quality of life and question 2 asked about patient’s overall perception of their health. The four domain scores denoted patient’s perception of quality of life in each particular domain. Domain scores were scaled in a positive direction (i.e. higher scores denote higher quality of life). The mean score of items within each domain were used to calculate the domain score [19].

### Assessment of selected cardio-metabolic biomarkers and blood pressure

All blood samples were collected after 12 h of overnight fasting at the day of examination. The lipid profile containing serum total cholesterol (TC), high density lipoprotein-cholesterol (HDL-C) and triglycerides (TG) was determined by enzymatic colorimetric methods (Roche Diagnostics Corp., Indianapolis IN) [20–24]. Low density lipoprotein-cholesterol (LDL-C) was calculated according to Friedewald et al. [25]. Disturbance in lipid profile was assessed according to *the National Health and Nutrition Examination Survey* [26]. The level of fasting blood glucose and glucose tolerance test were determined by the routine enzymatic method. The glucose metabolism was interpreted according to the *American Diabetes Federation* [27].

Blood pressure (BP) was measured during the study visit with a digital electronic tensiometer (Omron Corp., Kyoto, Japan) after a resting period of 10 min. The mean of three consecutive measurements were taken in the non-dominant arm with 3-min intervals between readings. Regular or large adult cuffs were used, depending on arm circumference of the examined patient. BP measurements were performed in accordance with the guidelines of the *European Society of Hypertension* [28].

### Nutritional assessment

The dietary intake was evaluated by 24 h dietary recall (Dietetyk, National Institute of Food and Nutrition, Warsaw, Poland). The local tables of food portion sizes and the weights of foods displayed in photos were used to estimate the amounts of food consumed. Several nutritional factors including total energy, carbohydrates, proteins, fats, fatty acids (saturated fatty acids-SFA, monounsaturated fatty acids-MUFA, polyunsaturated fatty acids-PUFA), selected vitamins (vitamin B1, B2, B6, B12, D and folate) were surveyed.

### Statistical approach

All statistical analyses were conducted with SAS Enterprise version 6.1. (SAS Institute Inc. USA). Single imputation was used to impute missing values using chained equations [29] by invoking Proc MI statement in SAS. Categorical variables were described using percentages and frequencies while continuous variables were described using mean and standard deviations. Statistical difference in background characteristics of study individuals were assessed using t-tests for continuous and Fischer’s exact test and Chi-square tests for categorical variables. Analysis of covariance (ANCOVA) was used to assess association of chronotype with quality of life domains and depression inventory. A similar method was also used to assess association between the quality of life domains and depression. ANCOVA assumptions of same slope across the groups were assessed by including the grouping variable along with covariates and the interaction term. Results did not indicate that there were interactions among covariates and the groups under study. Therefore, models without interaction were run for analysis. Age and sex-adjusted correlations were used to assess an association between quality of life domain and depression with objectively measured AHI, cardio-metabolic risk factors and nutrient intake. All analyses were adjusted for age and sex.

## Results

Baseline characteristics are presented in Table 1. Comparison between sexes showed that mean BMIs exceeded 30 kg/m2 in both groups. However, mean age was significantly higher in males, as well as the percentage of divorced males was higher in comparison to other marital statuses and females. There were no significant differences between alcohol drinks consumption, percentage of smokers, educational level, financial status and architecture of sleep between males and females. The mean AHI values exceeded 25 events per hour.

**Table 1:** Basic characteristics of studied patients with Obstructive Sleep Apnea (*n* = 64)

Analyzed variables	Males	Females	*p*-value
Number	59.4 (38)	40.6(26)	
Age (year)	60.3 ± 9.1	53 ± 12.1	0.01
Alcohol drinks consumption (g/day)	65.5 ± 46.4	63.5 ± 37.6	0.85
BMI (kg/m^2^)	31.3 ± 6.7	32.6 ± 5.5	0.38
Smokers (%)	66.36 (4)	63.64 (7)	0.99^e^
Education (%)			
Primary school	15.4 (4)	15.8 (6)	0.28
Highschool	65.4 (17)	47.4 (18)	
University degree	19.2 (5)	36.8 ( 14)	
Marital Status (%)			
Single	7.7 (2)	15.8 (6)	0.04^e^
Married	69.2 (18)	76.3 (29)	
Divorced	23.1 (6)	2.6 (1)	
Widowed	0 (0)	5.3 (2)	
Financial Status (%)			
Poor	11.5 (3)	15.8 (6)	0.42^e^
Good	84.6 (22)	71.1 (27)	
Very good	3.9 (1)	13.2 (5)	
AHI (events per hours)^a^	25.1 ± 25.7	25.4 ± 19.7	0.95
ODI (events per hours)^b^	26 ± 25.1	24.8 ± 20.2	0.85
Sleep architecture			
NREM Phase (%)^c^			
Stage of sleep N1	12.6 ± 12.8	14.1 ± 12.8	0.65
Stage of sleep N2	16.5 ± 9.3	16.8 ± 11.1	0.92
Stage of sleep N3	34.1 ± 10.9	30.5 ± 12	0.22
REM Phase (%)^d^			
Stage of sleep R	24.7 ± 12.2	23.7 ± 13.9	0.77

Data shown as: mean ± SD or % (number)

^a^AHI -Apnea/Hypopnea Index classification: normal (<5.0), mild (5.0–14.9), moderate (15.0–29.9), and severe (≥30.0 events per hour), ^b^ODI- oxygen desaturation index, ^c^NREM- no rapid eye movement ^d^REM-rapid eye movement expressed as percentage of total sleep, ^e^Fisher’s exact test

**Fig. 1:**
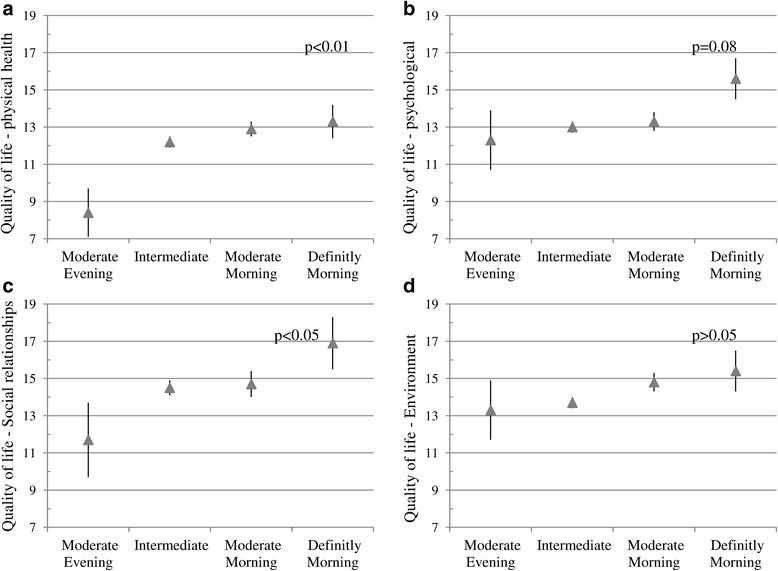
Association between quality of life domains (**a**. physical health, **b**. psychological, **c**. social relationship, **d**. environment) and chronotype in patients with Obstructive Sleep Apnea. *p* values for linear trend

**Fig. 2:**
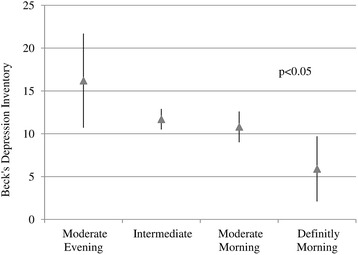
Association between chronotype and Beck’s depression inventory in patients with Obstructive Sleep Apnea. *p* values for linear trend

**Fig. 3:**
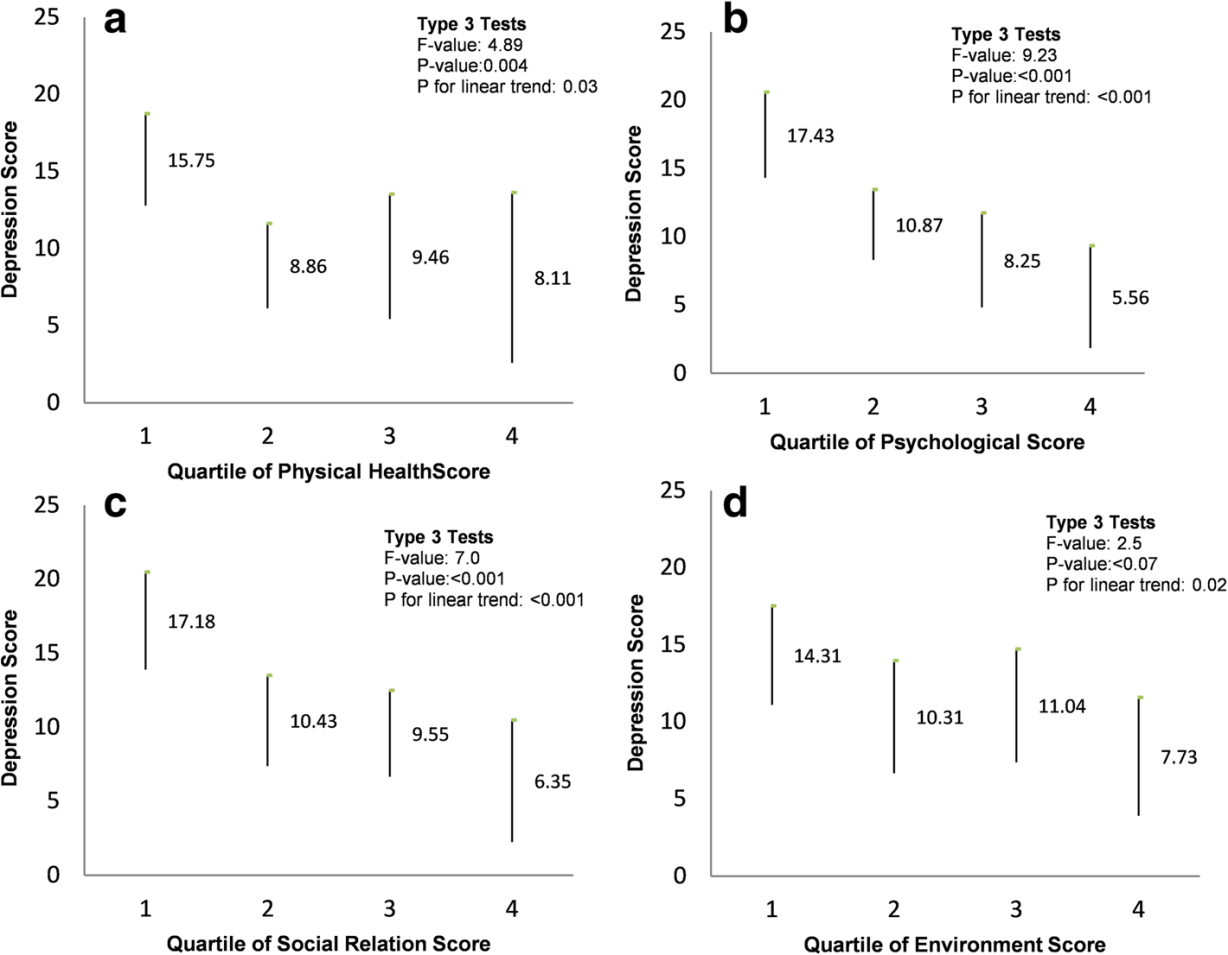
Association between quality of life domains (**a**. physical health, **b**. psychological, **c**. social relationship, **d**. environment) and Beck’s depression inventory in patients with Obstructive Sleep Apnea

Association of self-reported chronotype with quality of life and depression in study patients is shown in Table 2. Patients with morning type had significantly better physical health as compared to patient with intermediate or evening types. Other QOL domains including psychological, social relationship and environment however did not differed significantly between chronotype groups, there were significant liner trend for social health and near to significant trend for psychological domain (Fig. 1). Similarly there was a inverse linear significant decrease in depression score across groups from evening to absolute morning type (Fig. 2). Depression was significantly inversely associated with three of the four quality of life domains. Patients with better physical health, psychological and social relationships had significantly lower depression scores. There was a significant linear decrease in depression (score) across quartiles of increased (better) environment score (Fig. 3).

**Table 2:** Comparison of quality of life and depression inventory with morning/evening type in patients with Obstructive Sleep Apnea (*n* = 64)

Analyzed parameters	Self-assessment of chronotype^b^ Mean (SD)¶				*p*-ANCOVA
Main domains of Quality of Life^a^	Moderate Evening	Intermediate	Moderate Morning	Definitly Morning	
physical health	8.4 (1.3)	12.2 (0.3)	12.9 (0.4)	13.3 (0.9)	< 0.02
psychological	12.3 (1.6)	13 (0.3)	13.3 (0.5)	15.6 (1.1)	0.13
Social relationships	11.7 (2)	14.5 (0.4)	14.7 (0.7)	16.9 (1.4)	0.21
Environment	13.3 (1.6)	13.7 (0.3)	14.8 (0.5)	15.4 (1.1)	0.22
Depression inventory^c^	16.2 (5.5)	11.7 (1.2)	10.8 (1.8)	5.9 (3.8)	0.24

¶Data shown as adjusted means (SD) for age and sex

^a^WHOQOL-BREF, ^b^Morningness-eveningness questionnaire, ^c^Beck’s Depression Inventory

Diastolic blood pressure was inversely correlated with psychological and social relationships QOL domains. Moreover, higher concentration of HDL-C was positively correlated with the environment QOL domain and morning chronotype. Furthermore, the concentration of TG and fasting glucose was positively correlated with social relationships and environment –QOL domains, while the intake of fat, MUFA and SFA was inversely correlated with morning chronotype. Intake of proteins and vitamin B6 was significantly positively related to better social relationships (Table 3).

**Table 3:** Correlation of Quality of Life Domains, depression, morningness-eveningness types and Apnea Hypopnea Index, selected cardio-metabolic biomarkers and nutrients (adjusted for age and sex; Pearson Partial Correlation Coefficients, *n* = 64) in in patients with Obstructive Sleep Apnea

Analyzed variables	Quality of life				Depression inventory	Morningness-eveningness
	Physical health	Psychological	Social relationships	Environment		
AHI (events/hours)^a^	0.11 (0.43)	0.13 (0.33)	0.07 (0.59)	0 (0.98)	0.11 (0.4)	0.15 (0.27)
SBP (mmHg)^b^	−0.02 (0.88)	−0.03 (0.85)	−0.12 (0.36)	0.19 (0.15)	−0.06 (0.65)	0.13 (0.34)
DBP (mmHg)^c^	−0.25 (0.06)	−0.36 (0.01)	−0.29 (0.03)	0.01 (0.93)	0.17 (0.2)	0.05 (0.72)
TC (mmol/dl)^d^	0.02 (0.86)	0.28 (0.04)	0.05 (0.73)	0.23 (0.08)	−0.03 (0.8)	−0.09 (0.52)
HDL (mmol/dl)^e^	−0.05 (0.7)	0.02 (0.88)	−0.12 (0.38)	0.25 (0.06)	−0.04 (0.77)	0.03 (0.84)
LDL (mmol/dl)^f^	0.06 (0.68)	0.3 (0.02)	−0.02 (0.89)	0.14 (0.31)	−0.01 (0.94)	−0.08 (0.57)
TG (mmol/dl)^g^	0.03 (0.82)	0.11 (0.4)	0.27 (0.04)	0.03 (0.8)	0.04 (0.75)	−0.14 (0.28)
Fasting glucose (mmol/dl)	0.03 (0.81)	−0.08 (0.57)	0.03 (0.85)	−0.1(0.45)	0.3 (0.02)	0.12 (0.37)
Energy (kcal)	−0.09 (0.49)	−0.04 (0.76)	0.23 (0.09)	0.08 (0.54)	−0.06 (0.67)	−0.13 (0.32)
Carbohydrates (g)	0.01 (0.95)	0.02 (0.88)	0.2 (0.14)	0.18 (0.18)	−0.04 (0.75)	0.05 (0.71)
Protein (g)	−0.2 (0.13)	−0.12 (0.39)	0.27 (0.04)	−0.01 (0.93)	−0.08 (0.55)	−0.14 (0.3)
Fat (g)	−0.11 (0.39)	−0.07 (0.59)	0.17 (0.21)	−0.03 (0.83)	−0.05 (0.68)	−0.28 (0.03)
PUFA (g)^h^	−0.11 (0.39)	−0.15 (0.27)	0.09 (0.52)	−0.12 (0.38)	−0.08 (0.56)	−0.11 (0.43)
MUFA (g)^i^	−0.08 (0.53)	−0.03 (0.85)	0.19 (0.14)	0.01 (0.96)	−0.02 (0.86)	−0.3 (0.02)
SFA (g)^j^	−0.12 (0.39)	−0.07 (0.6)	0.13 (0.35)	−0.01 (0.92)	−0.06 (0.63)	−0.27 (0.04)
Vit B1 (mg)	0.11 (0.4)	−0.01 (0.97)	0.13 (0.32)	0.19 (0.16)	−0.12 (0.37)	−0.02 (0.89)
Vit B2 (mg)	−0.18 (0.19)	−0.03 (0.8)	0.19 (0.15)	−0.06 (0.63)	−0.09 (0.52)	−0.08 (0.56)
Vit B6 (mg)	0.06 (0.65)	−0.06 (0.64)	0.27 (0.04)	0.15 (0.27)	−0.13 (0.34)	0.05 (0.69)
Vit B12 (ug)	−0.06 (0.67)	0.06 (0.63)	0.13 (0.33)	−0.01 (0.95)	−0.03 (0.82)	−0.27 (0.04)
Folate (ug)	0.05 (0.69)	0.05 (0.71)	0.2 (0.14)	0.08 (0.54)	−0.2 (0.13)	0.18 (0.17)
Vit D (ug)	0.05 (0.68)	0.09 (0.49)	0.12 (0.36)	0.06 (0.64)	−0.09 (0.49)	−0.25 (0.06)

Data expressed as adjusted correlation coefficient (*p*-value)

^a^AHI -Apnea/Hypopnea Index classification: normal (<5.0), mild (5.0–14.9), moderate (15.0–29.9), and severe (≥30.0 events per hour).)^b^SBP- systolic blood pressure,)^c^DBP-diastolic blood pressure,^d^TC-total cholesterol, ^e^HDL-high density lipoprotein, ^f^LDL- low density lipoprotein, ^g^TG- triglycerides, ^h^PUFA- polyunsaturated fatty acids, ^i^MUFA- monounsaturated fatty acids, ^j^SFA-saturated fatty acids

## Discussion

Here we present that the morning chronotype positively influence while depression negatively influence QOL in OSA patients. In addition, QOL, depression and chronotype were correlated with HDL-C, TG, fasting glucose and dietary intakes of fat, MUFA, SFA and vitamin B12.

Gender differences may exist in the clinical signs and PSG of OSA. It has already been shown that men experience OSA at two to three times the rate of women [30], present with OSA at a younger age [31], and have a higher AHI [32] than women [6]. However, in the current study we did not find differences in the mean value of AHI index and sleep architecture between sexes. Moreover, all included patients were characterized by obesity, which has commonly been reported by other authors [33]. Most of the patients suffering from OSA self-report poor sleep (even up to 82 %) [34]. Further, a poor sleep status in OSA patients is usually associated with fatigue and may result in a compensatory increase in caloric intake leading to weight gain. As we have examined mostly obese patients, the correlation between diastolic blood pressure and QOL was not surprising.

The incidence of depressive disorders, especially in OSA patients, is widely investigated, and it may reflect changes in the sources of fat in the Western diet [35]. Usually, PUFA or MUFA are replaced by SFA and trans fatty acids (TFA), which was reported as a relevant risk factor for depression [36, 37]. In the current study, higher fat intake and MUFA was correlated with morning type of patients that present better physical health and social relationships of QOL. Nevertheless, little is still known about dietary patterns focusing specifically on subtypes of fat intake in OSA patients and their relationship with the risk factors of depression. Clinical studies have already been shown that olive oil consumption (being a good source of MUFA) improves depression and mood scores which could be also associated to an increased amount of total fat intake being protective for the development of depressive symptoms [38–40]. As reported by Lang et al [41], selected nutritional compounds, e.g. vitamins from B group, have been postulated to be used as “ad-on strategies” in anti-depressant treatment. Moreover, due to the observed higher level of homocysteine, involved in the development of dyslipidemia, in OSA patients, the role of vitamins B6 and B12 seems to be especially interesting [42]. Further, it was not surprising for us that the intake of these nutrients was positively related to better QOL, especially pronounced in morning type of patients. The positive correlations between the level of TG and fasting glucose with social relationship and environment of QOL may suggest the role of social life as one of the key element in patient’s health. The cardiovascular, neurocognitive, and metabolic manifestations of OSA can have a significant impact on patient health and QOL, therefore self-estimation of QOL in relation to nutrition and depression is of high importance [43]. A random sampling of middle-aged workers in the Wisconsin Sleep Cohort Study [29] has shown that 22.6 % of females and 15.5 % of males with an AHI > 5 reported experiencing frequent unrefreshing sleep and uncontrollable sleepiness that interfered with life.

### Limitation

We had applied the self-reported assessment tools (questionnaires) for the evaluation of QOL and depression. However, the chronotype was assessed by both objective methods (PSG) and morningness-eveningness questionnaire, to identify patients with OSA which gives better characteristic of the study population. Further, it is very commonly observed that obese individuals underreport their dietary intake, which could also influence obtained results, especially considering that 24 h dietary recalls were applied. However, in this case, the correlations that were found may support stronger relationships between nutrition and other analyzed factors showing the importance of dietary counselling in this specific group of patients.

## Conclusions

In conclusion, both chornotype and depression influence QOL in OSA patients where morning type is associated with better physical health and social relationships and increase in depression index deteriorate physical health, psychological and social relationship QOL domains. QOL as well as depression and chornotype are also influenced by selected cardio-metabolic factors and dietary intake.
